# Vegetation assessments under the influence of environmental variables from the Yakhtangay Hill of the Hindu-Himalayan range, North Western Pakistan

**DOI:** 10.1038/s41598-022-21097-4

**Published:** 2022-12-05

**Authors:** Hameed Ullah, Shujaul Mulk Khan, Mariusz jaremko, Sadia Jahangir, Zahid Ullah, Iftikhar Ali, Zeeshan Ahmad, Hussain Badshah

**Affiliations:** 1 Department of Plant Sciences, Quaid-i-Azam University, Islamabad, 45320 Pakistan; 2 Member Pakistan Academy of Sciences, Islamabad, Pakistan; 3 Smart-Health Initiative (SHI) and Red Sea Research Center (RSRC), Division of Biological and Environmental Sciences and Engineering (BESE), King Abdullah University of Science and Technology (KAUST), Thuwal, 329555-6900 Saudi Arabia; 4 Mirpur University of Science and Technology (MUST), Azad, Jammu and Kashmir Pakistan; 5 State Key Laboratory of Molecular Developmental Biology, Institute of Genetics and Developmental Biology, Chinese Academy of Science, Beijing, 100101 China; 6 Centre for Plant Science and Biodiversity, University of Swat, Charbagh, 19120 Khyber Pakhtunkhwa Pakistan

**Keywords:** Ecology, Plant sciences

## Abstract

Vegetation structures and dynamics are the result of interactions between abiotic and biotic factors in an ecosystem. The present study was designed to investigate vegetation structure and species diversity along various environmental variables in the Yakhtangay Hills of the Hindu-Himalayan Mountain Pakistan, by using multivariate statistical analysis. Quadrat quantitative method was used for the sampling of vegetation. PC-ORD version 5 software was used to classify the vegetation into different plants communities using cluster analysis. The results of regression analysis among various edaphic variables shows that soil organic matter, total dissolved solids, electrical conductivity, CaCO_3_ and moisture contents shows a significant positive correlation with species abundance, while the soil pH has inverse relationship with plant species abundance. Similarly, species richness increases with increase in soil organic matter, CaCO_3_ and moisture contents, while decrease with increase in soil pH, total dissolved solids and electrical conductivity (*p* < 0.05). The vegetation was classified into four major plant communities and their respective indicators were identified using indicator species analysis. Indicator species analysis reflects the indicators of the study area are mostly the indicators to the Himalayan or moist temperate ecosystem. These indicators could be considered for micro-habitat conservation and respective ecosystem management plans not only in the study area but also in other region with similar sort of environmental conditions.

## Introduction

Environmental gradients are those components of the environment which influence vegetation composition and structure either directly or indirectly^1,2^. Species that have significant environmental tolerance developed a distinct plant associations with unique floristic and structural attributes^3^. Vegetation of an area can be classified or categorized into different communities/associations based on various similarities and differences under the influence of different environmental gradients^4^. Floristic diversity is usually dependent upon environmental factors including topography, soil and climate. Various plants life characteristics such as leaf and life forms (biological spectra), phenological attributes, further mirrors the existence of ecological and natural surrounding^5^. Whereas, vegetation is the expression of environment inside the specified locality within a specified period, therefore it needs to be properly studied along with it is surrounding both at species and at community level^6^. The term vegetation indicates the life of plant in a specific region, while flora represents plant composition. Analyzing the characteristics, classification, relationship and distribution of plant communities and the effort to describe the diversity of species inside the plant communities is termed as phytosociology. The primary aim of the phytosociology is describing the vegetation structure and classification by a meaningful way through quantitative method^7,8^.

The limitation of resources inside the environment affect the plant community properties, structure, species distribution, and ecosystem functions like productivity and nutrient circulation. Though till now a couples of factors has been recognized and a few studies have investigated a single plant or a combined response of a community to such limitations^9^. Due to the lack of these information which may be concerned to many treatments required to evaluate such responses to both single resource restrictions and their interrelationship with other factors. Each species inside a community might be limited due to the availability or lack of different resources, making community level and individuals plant responses in species-rich communities difficult to interpret^10^. Despite these challenges, such insight is crucial to understand how individual’s species and communities are affected by natural and anthropogenic disturbances^11^. Thus, it is crucial to understand the vegetation distribution pattern and influencing factors at different scales^12,13^. It is very important to understand the vegetation-environment relationship to check the effect of changes in environment^14^. It has been observed by various plant ecologist that not only at local or regional scale, the diversity of plants may be influenced by abiotic conditions, but also depend on other ecosystem processes, such as biotic interaction and seed dispersal limitations^15,16^. The limiting factors inside any biome, limit the total number and diversity of the species. While other environmental gradients, particularly topography, elevation, snow cover, soil moisture and pH, have been analyzed and concluded that these are correlated with plant species richness at both regional and local levels^17–19^.

Among the abiotic gradients, topography contributing to the physiognomic differentiation for vegetation and thus play a significant role in the heterogeneity of habitat^20–23^ and consequently changing the structure of plant community and their species composition. An extensive variation in mountain landscape variation regarding to the soil age, erosion rates, topography, hydrology, among other factors has effects the structure, composition and ultimately function of the ecosystem^24–27^. Landscape matrices are also molded by various topographic element, such as altitude, aspect, and slope^28^. Therefore, it is important to investigate the vegetation at multiscale rather than through a single scale, to fulfill different needs for resource management. Altitudinal gradient also affect vegetation structure and species composition of different ecosystem and various studies indicated that plants diversity is highest at intermediate elevation, like in tropical rain forests^29–32^. Moreover, a humped (unimodal) curve between species diversity, and the increase in altitude was observed at Himalayan Mountain^23^.

Among the edaphic factors, the soil nutrients play an important role influencing certain plants parameters such as tree height, basal area, canopy cover and consequently influencing the structure of plant communities^33,34^. Several studies have been carried out to check the relationship between the change in species richness and the availability of soil gradients^35,36^. Generally lower species richness was observed at lower nutrient level, that increases with the intermediate level of the nutrients while gradually decline at high nutrient level making a typical “humped back curve” in response to the nutrient gradient.

This pattern has been found by a numbers of plants ecologist^37,38^. Soil pH is one of the basic environmental gradients suggested to be an important driver of the number of vascular species at the local scale (fine-scale species richness, alpha diversity)^39^. It has been suggested that on acidic pH (pH < 4), the number of species could be constrained by high phytotoxicity ^40–43^ and limit nutrition availability. It has been observed that on high-pH soils (pH > 7), phosphorus and iron become limitedly available nutrients because of their decrease in solubility and thus affecting on species diversity of an area. Slope facing to poles are wetland cool, with rich organic matter and deeper soil and characterized by mesic vegetation, while the slope facing toward equator are usually hot and xeric type with low soil nutrients and thus having severe erosion, subsequently they are supporting xeric type of vegetation^44,45^. It has a common observation that sunny slope retains less moisture compared to shady slope, because of greater solar radiation and high evaporation. Therefore, the vegetation of the sunny slope such as grasses must be xeric type, drought tolerating and radiation resistant. On the other hand, shade loving plant such as forbs are dominant on shady slopes. Soil moisture availability is a key driver for both biological and chemical processes and one of the important limiting factors responsible for the productivity of plant^46,47^. Owing to the response, vegetation type and structure and type can influence soil water dynamics and soil characteristics including soil hydraulic characteristics^48–50^. Taking water as a source of nutrients if measured generate a similar humped shaped curve with vegetation. Usually, plant species richness shows a positive logarithmic relationship with rainfall^51,52^. The geological conditions, seawater, and domestic activities (domestic, agricultural, and industrial effluents) are the prime source of the particulate matters^53^. Due to the rapid changes in landscape, climate and geo-climate history mountain ecosystem possess diverse biological communities all over the world.

Therefore, this study was designed to analyze vegetation of the Yakhtangay hill, Hindu-Himalayan series of Pakistan in relation to various physiographic and edaphic variables. It is hypothesized that slope, altitude, and aspect of the mountain plays a significant role in vegetation structure and species diversity. We aim to analyze plants assemblage i.e., communities’ formation under the influence of environmental variables including edaphic and climatic variables on species diversity i.e., species abundance and richness by using recently developed analytical techniques. This study will answer how to classify and identify indicator species of particular habitat or community.

## Materials and methods

### Study area

**Figure 1:**
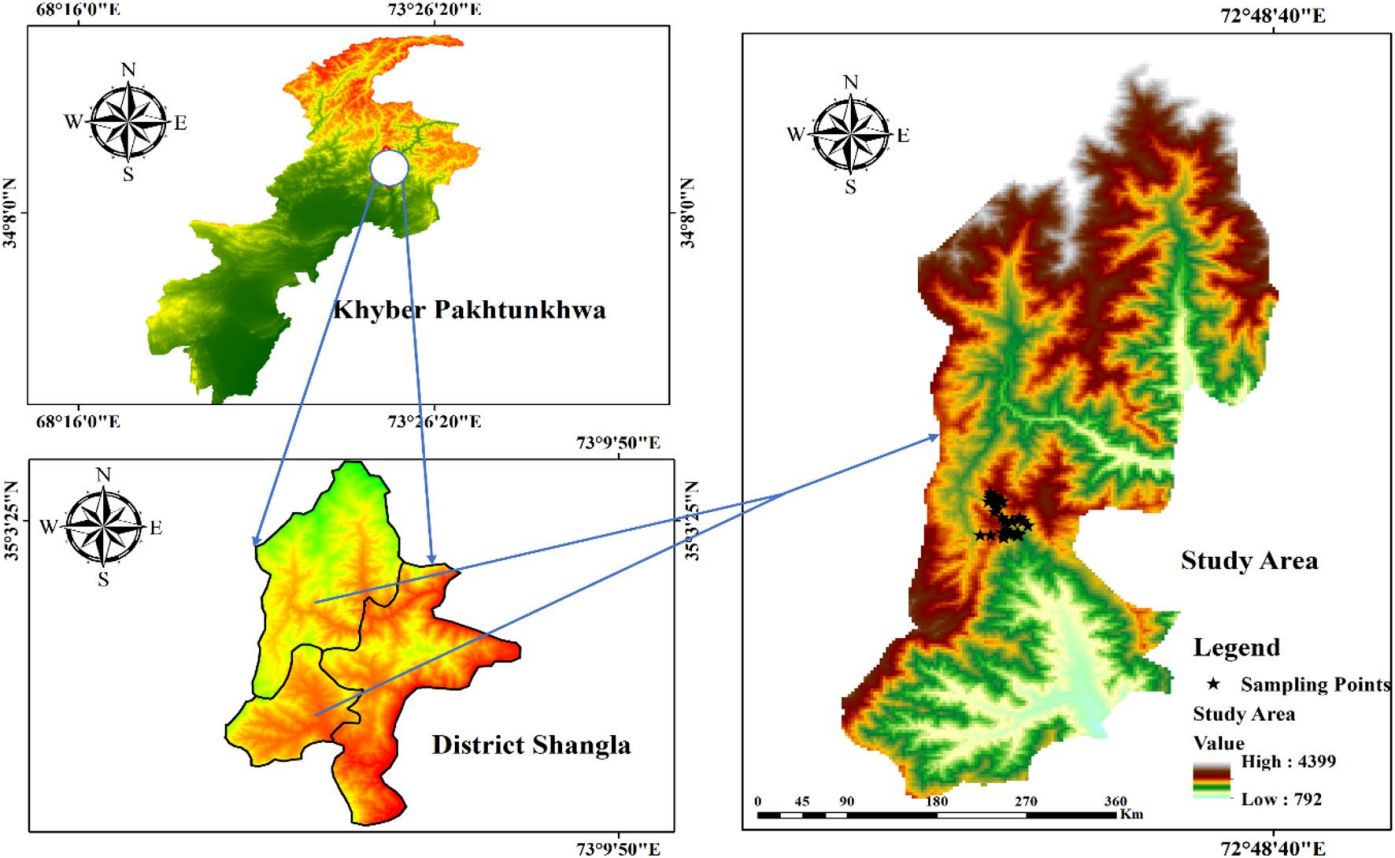
GIS generated map of the study area representing the sampling localities^56^.

District Shangla is located at western extremities of the Himalayan and eastern Hindu Kush Mountain series generally termed as Hindu-Himalayas, in the Khyber Pakhtunkhwa province of Pakistan. Topographically, it consists of narrow valleys and high mountains covered by dense forest at elevation range from 1300 to 3000 masl^54^. The Yekhtangay hill is one of such high mountains famous tourist spots, located at 34°-31' to 33°-08' north latitude and 72°-33' to 73°-01 south longitude at about 2200 masl. Temperature of the study area observed severe cold in winter and moderate in summer. The area is covered mostly by snow, usually from November to April that makes winter season more prolonged than summers. Mountains of the study area possess diverse moist temperate forest. The inhabitants in the foothill of Yakhtangay hills are dependent on the forest resources as source of earning, agricultural tools and for fuel purposes. Therefore, the forest of the area is under severe anthropogenic pressure. The soil of the area is mostly clay suitable for the growth of diverse vegetation (Fig. 1, Table 1; Supplementary Table S1)^55^.

**Table 1:** Altitudinal ranges and coordinates data of the selected sampling locations. (*T* transect, *Q* quadrat, *W* west, *N* north, *E* east)

S. no	Quadrat name	Altitude (feet)	Latitude	Longitude
1	T1Q1W	6157	34° 50.401' N	72° 37.295' E
2	T1Q2W	6277	34° 50.471' N	72° 37.317﻿' E
3	T1Q3W	6406	34° 50.316' N	72° 37.395'﻿ E
4	T1Q4W	6856	34° 288.316' N	72° 37.250'﻿ E
5	T2Q1W	6242	34° 50.317' N	72° 37.423'﻿ E
6	T2Q2W	6530	34° 50.408' N	72° 37.429'﻿ E
7	T2Q3W	6714	34° 50.318' N	72° 37.443'﻿ E
8	T2Q4W	6960	34° 50.423' N	72° 37.399'﻿ E
9	T2Q5W	7206	34° 50.50' N	72° 37.405'﻿ E
10	T2Q6W	7511	34° 50.401' N	72° 37.363'﻿ E
11	T3Q1W	6316	34° 50.126' N	72° 37.462'﻿ E
12	T3Q2W	6515	34° 50.123' N	72° 37.462'﻿ E
13	T3Q3W	6813	34° 50.317' N	72° 37.569'﻿ E
14	T3Q4W	7216	34° 50.442' N	72° 37.413'﻿ E
15	T3Q5W	7419	34° 50.254' N	72° 37.474'﻿ E
16	T4Q1W	6311	34° 50.334' N	72° 37.614'﻿ E
17	T4Q2W	6611	34° 50.316' N	72° 37.617'﻿ E
18	T4Q3W	6844	34° 50.311' N	72° 37.546'﻿ E
18	T4Q4W	6930	34° 50.312' N	72° 37.411'﻿ E
19	T4Q5W	7456	34° 50.298' N	72° 37.398'﻿ E
20	T5Q1W	6411	34° 50.696' N	72° 37.319'﻿ E
21	T5Q2W	6823	34° 49.516' N	72° 37.694'﻿ E
22	T5Q3W	7411	34° 49.630' N	72° 37.611'﻿ E
23	T5Q4W	7887	34° 49.614' N	72° 37.315'﻿ E
24	T6Q1W	6530	34° 49.703' N	72° 38.034'﻿ E
25	T6Q2W	6914	34° 49.714' N	72° 38.037'﻿ E
26	T6Q3W	7190	34° 49.713' N	72° 38.035'﻿ E
27	T6Q4W	7494	34° 49.711' N	72° 37.034'﻿ E
28	T6Q5W	7641	34° 49.718' N	72° 38.038'﻿ E
29	T6Q6W	7888	34° 49.712' N	72° 38.037'﻿ E
29	T7Q1W	6510	34° 49.503' N	72° 38.039'﻿ E
30	T7Q2W	6930	34° 49.411' N	72° 38.040'﻿ E
31	T7Q3W	7240	34° 49.302' N	72° 38.061'﻿ E
32	T7Q4W	7530	34° 49.201' N	72° 38.081'﻿ E
33	T7Q5W	7940	34° 49.411' N	72° 38.091'﻿ E
35	T1Q1E	6019	34° 49.413' N	72° 38.475'﻿ E
36	T1Q2E	6613	34° 49.411' N	72° 38.466'﻿ E
37	T1Q3E	6856	34° 49.410' N	72° 38.466'﻿ E
38	T2Q1E	6113	34° 49.414' N	72° 38.215'﻿ E
39	T2Q2E	6443	34° 49.417' N	72° 38.219'﻿ E
40	T2Q3E	6711	34° 49.422' N	72° 38.224'﻿ E
41	T2Q4E	7244	34° 49.425' N	72° 38.219'﻿ E
42	T3Q1E	6244	34° 49.102' N	72° 38.118﻿' E
43	T3Q2E	6516	34° 49.105' N	72° 38.113'﻿ E
44	T3Q3E	6881	34° 49.108' N	72° 38.116'﻿ E
45	T3Q4E	7216	34° 49.109' N	72° 38.119'﻿ E
46	T3Q5E	7519	34° 49.111' N	72° 38.119'﻿ E
47	T4Q1E	6115	34° 49.094' N	72° 38.011'﻿ E
48	T4Q2E	6416	34° 49.095' N	72° 38.014'﻿ E
49	T4Q3E	6732	34° 49.097' N	72° 38.015'﻿ E
50	T4Q4E	6994	34° 49.096' N	72° 38.011'﻿ E
51	T5Q1E	6219	34° 50.112' N	72° 39.114'﻿ E
52	T5Q2E	6433	34° 50.113' N	72° 39.117'﻿ E
53	T5Q3E	6611	34° 50.115' N	72° 39.113'﻿ E
54	T5Q4E	6713	34° 50.116' N	72° 39.111'﻿ E
55	T5Q4E	6814	34° 50.114' N	72° 39.114'﻿ E
56	T5Q5E	7124	34° 50.107' N	72° 39.119'﻿ E
57	T5Q6E	73,144	34° 50.102' N	72° 39.117'﻿ E
58	T1Q1W	6157	34° 50.401' N	72° 37.295'﻿ E
59	T1Q2W	6277	34° 50.471' N	72° 37.317'﻿ E
60	T1Q3W	6406	34° 50.316' N	72° 37.395'﻿ E

### Data collection

The plants data of summer and spring were collected during the months of July–August 2020 and April-May 2021, respectively. Transect and quadrat quantitative methods were applied for the sampling of vegetation^57^. Permission was taken from the local forest and wildlife department for carrying out plants collection activities in the forest. Quadrats were placed systematically along each transect at 200 m intervals using Global Positioning System (GPS). Quadrats having size of 20 × 20 m, 10 × 10 m and 2 × 2 m were taken place for tree, shrubs and herb species, respectively^58^. Quantitative data for each plant species were recorded to measure the phytosociological attributes including total frequency, density and cover. Plant specimens were collected and identified with help of Flora of Pakistan and expert taxonomist^59^. The dried specimens were preserved in the Herbarium of Pakistan, Quaid -i-Azam University, Islamabad, Pakistan. The biological spectra such as leaf form and life from were determined for each plant species using Raunkiaer^60^ classification.

### Phytosociological attributes/quantitative characteristics of the vegetation

The quantitative characteristics of vegetation such as density, relative density, abundance, cover, relative cover, frequency, relative frequency, and importance value index were calculated by using the following various formulas, followed by^61,62^.

$${\text{Density }}\left( {\text{D}} \right)\, = \frac{{Total\,number\,of\,individuals\,of\,a\,species\,in\,the\,sampled\,plot}}{{Total\,sampled\,area\,}}$$

$${\text{Relative Density}} = \left( {{\text{RD}}} \right) \, = \frac{{Density\,of\,an\,individual\,species\,in\,a\,quadrat\,\,}}{{Total\,density\,of\,all \,individuals\,or\,plants\,\,\,}} \times 100$$

$${\text{Cover }} = \frac{{Total\,cover \,of \, \,a\ species}}{Total \,sampled \,area}$$

$${\text{Relative Cover }}\left( {{\text{RC}}} \right) = \frac{{ \,Cover\,of\, an\, individual\, plant\, species}}{Total \,cover \,of \,all \,plant\, species} \times 100$$

$${\text{Frequency }}\left( {\text{F}} \right) \, = \frac{{Number\,of\,quadrats\,of\,occurance\,of\,a\,species}}{{Total\,numbers\,of\,quadrats\,taken\,for \,sampling}} \times 100$$

$${\text{Rative Frequency }}\left( {{\text{RF}}} \right) \, = \frac{Frequency \,of\, a\, species}{{Total\,frequency\, of\, all \,species}} \times 100$$

$${\text{Importance Value Index }}\left( {{\text{IVI}}} \right) = \frac{RD + RF + RC}{3}$$

### Topographic and climatic information

The topographic and climatic factors such as altitude, aspect, slope angle, availability of sunlight, moisture and soil characteristics greatly influence vegetation structure, species composition and community structure in one or other way. Topographic gradients of the area such as altitude, elevation, height, slope angle, aspect were measured through the GPS essential application. The altimeter was used to record the altitude data, while a compass was used to check north, south, east, and west aspect of the mountain.

### Soil analysis

Soil samples were collected from 1 to 15 cm depth with the help a digger. About 20–25 g of soil samples from three different spots of each quadrat were collected and homogenized into one sample. A specific tag number was labeled according to the specific quadrat^63^. Various analysis of the soil were performed including soil moisture contents, texture, organic matter, calcium carbonates, Electrical Conductivity (EC), Total Dissolved Solids (TDS) and soil pH using different standard protocols^48,64,65^.

### Statistical data analysis

PC-ORD (version 5) software was used to classify the plant species and stations/quadrat into potential plant communities based on their similarities and differences^66^. Indicator species were identified using Indicator Species Analysis (ISA). While CANOCO software (version 4.5) was used for canonical correspondence analysis to investigate the relationship between vegetation and environmental variables^67–69^. Regression analysis was used for the assessment of the relationship between species richness/abundance along with various environmental variables using R Software (https://cran.r-project.org/bin/windows/base/)^70^.

## Results

### Floristic composition and biological spectra of the vegetation in the study area

A total of 114 plant species belong to 45 families were recorded from the studied region. Rosaceae was recorded as dominant family followed by Poaceae and Asteraceae (Supplementary Table S2). The dominant species were *Pinus wallichiana* (IVI = 2367), *Viburnum grandiflorum* (IVI = 2118), *Buxus wallichiana* (IVI = 1697) and *Bistorta amplexicaulis* (IVI = 450) (Supplementary Table S3). While among the leaf form, microphylls was the dominant (24 species that constituents about 33% of the total plants), followed by leptophylls (24%) and nanophyll (16%). Hemicryptophytes and nanophanerophytes were the dominant life forms that constituents about 52% of the total plant species. While therophytes constituents about 20% followed by mesophanerophytes (8%) in the region.

### Impact of edaphic factors on plant species abundance

**Figure 2:**
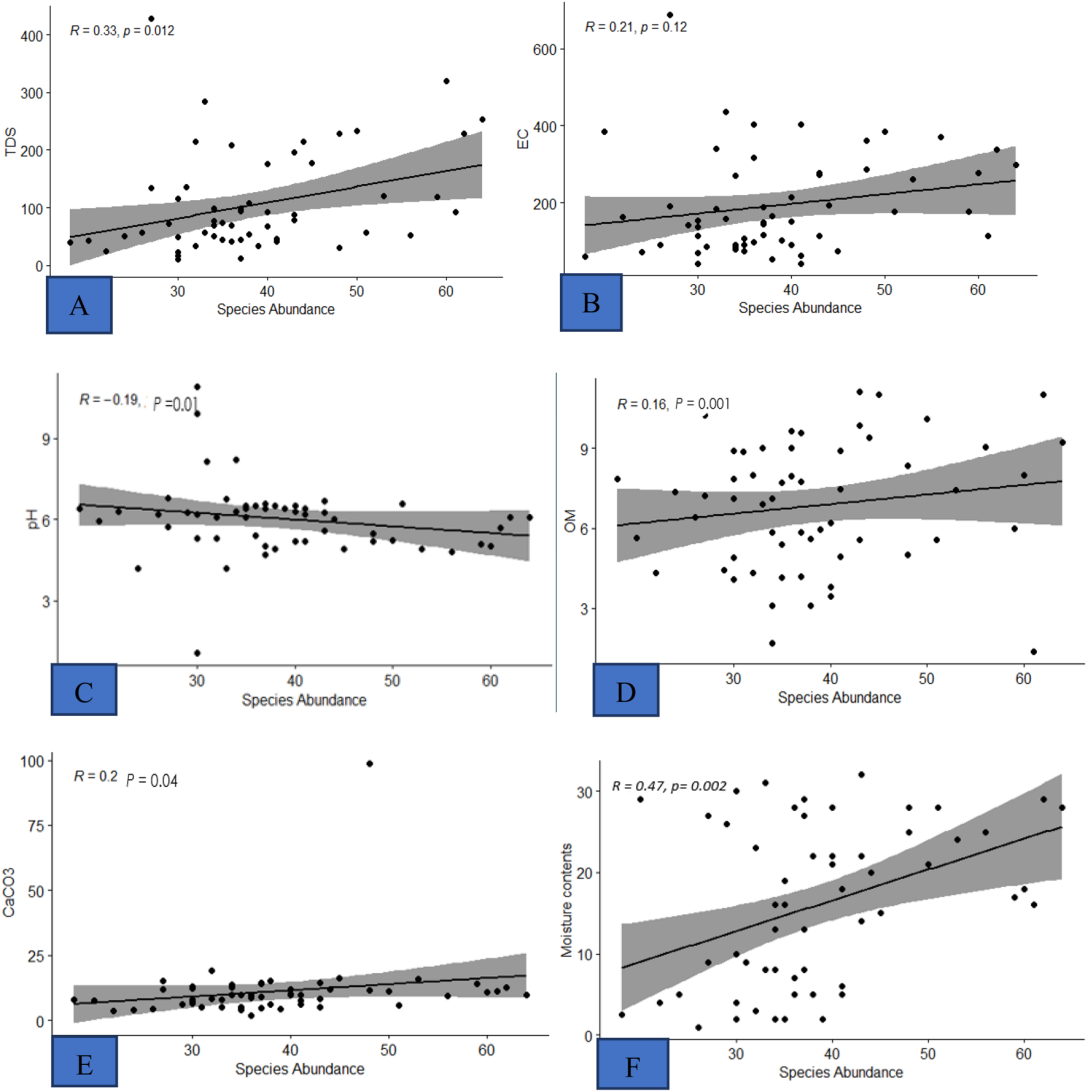
Regression analysis between soil variables and plant species abundance. Impact of soil (**A**) TDS (**B**) EC (**C**) pH (**D**) OM (**E**) CaCO_3_ and (**F**) moisture contents with species abundance.

Impact of edaphic factors on plant species were analyzed to check whether these variables are significantly representing the vegetation abundance of the area or not. The data of edaphic variables and species abundance were analyzed in” R” software version 4.0.4 through regression analysis. The result showed that the soil organic matter, TDS, EC, CaCO_3_ and moisture contents have a linear relationship (i.e., positive correlation) with species abundance (with degree of correlation, R = 0.16, R = 0.33, R = 0.21, R = 0.20, R = 0.47, respectively) at significant level (p value < 0.05). While the relationship between soil pH and plant species abundance represent a negative correlation with R = − 0.19 and p value 0.001 (Fig. 2).

### Impact of edaphic factors on species richness

**Figure 3:**
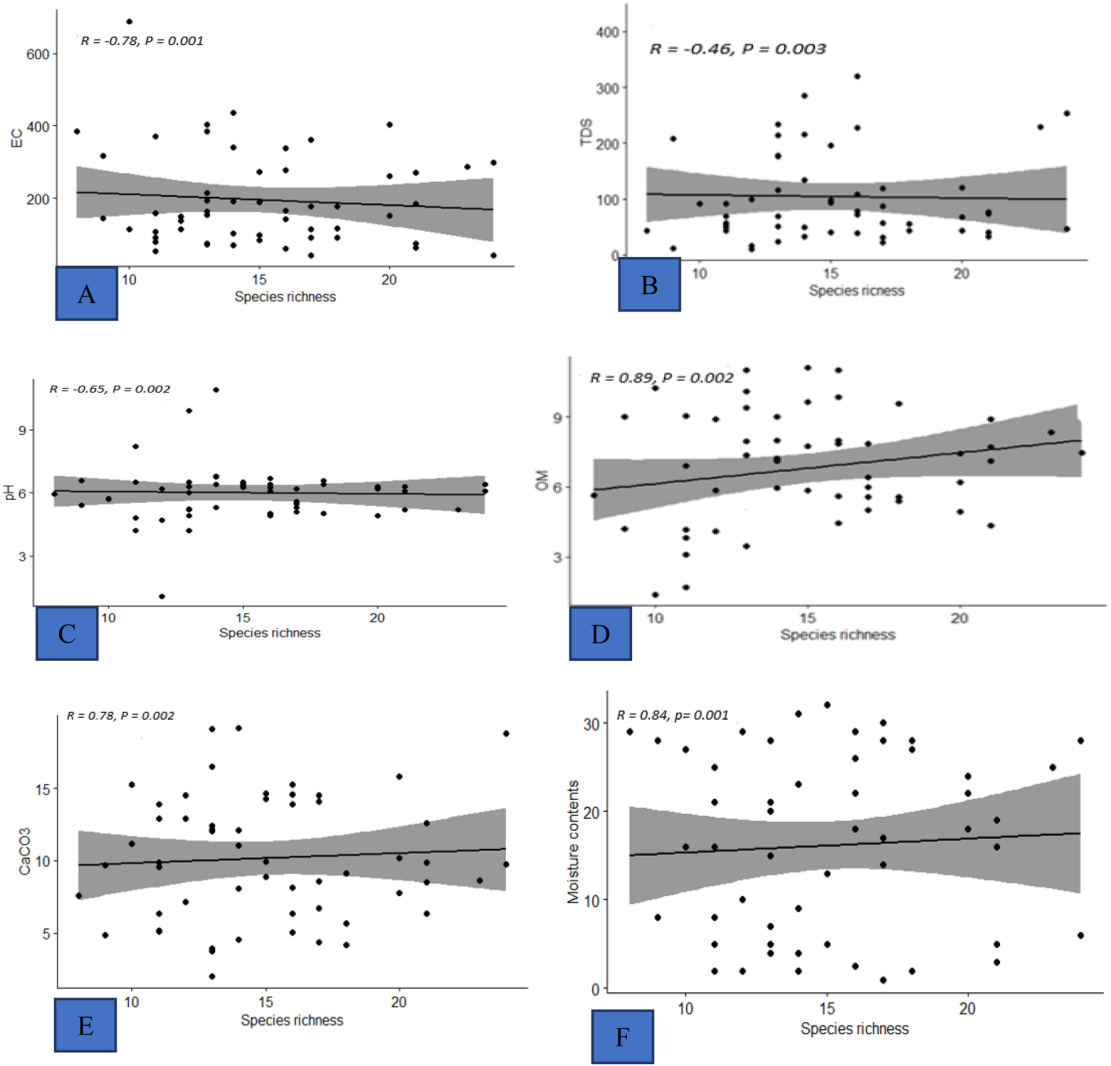
Regression analysis between soil variables and plant species richness. Impact of soil (**A**) EC (**B**) TDS (**C**) pH (**D**) OM (**E**) CaCO_3_ (**F**) moisture contents with species richness . Here, *OM* organic matter, *EC* electrical conductivity, *TDS* total dissolved solvents.

The regression analysis between species richness and edaphic factors shows that soil pH, TDS and EC are negative correlated with species richness having beta values i.e., R = − 0.65, R = − 0.46 and R = − 0.78, respectively at p value < 0.05 significance level. The relationship between soil organic matter (R = 0.89), calcium carbonates (R = 0.78) and moisture contents (R = 0.84) were positively correlated with species richness at, p-value < 0.05 (Fig. 3).

### Species area curve

**Figure 4:**
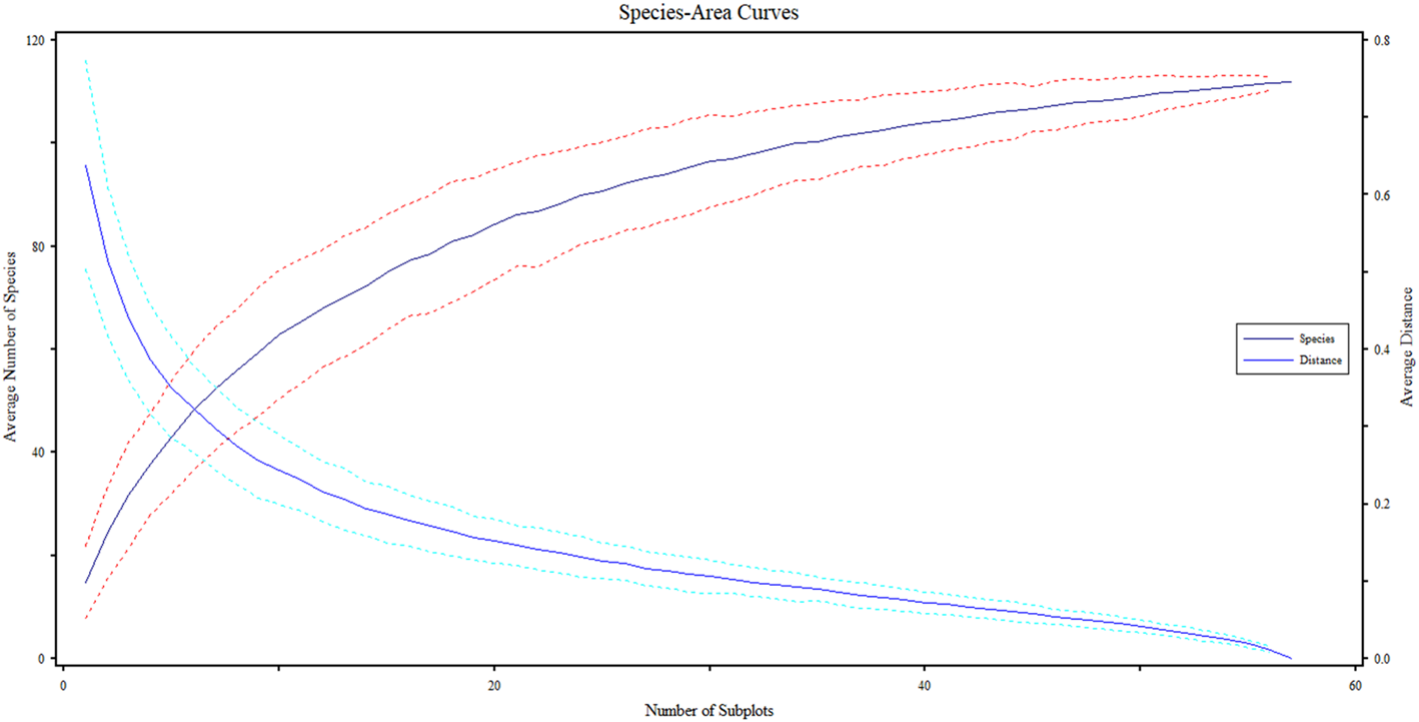
Species area curves representing relationship between occurring of new plant species along the sampling quadrats through compositional area curves.

We used the PC-ORD version 5 software to find that either the quadrat size was adequate or not through abundance data combined with Sorenson distance value in the form of species area curve and compositional area curve^61^. The result shows that the quadrat number 10 shows maximum numbers of plant species appearing and are continued up to quadrat number 40, then declines till the last quadrat with minimum number of species illustrating the adequacy of the sample size (Fig. 4).

### Formation of plant communities through cluster analysis (CA)

**Figure 5:**
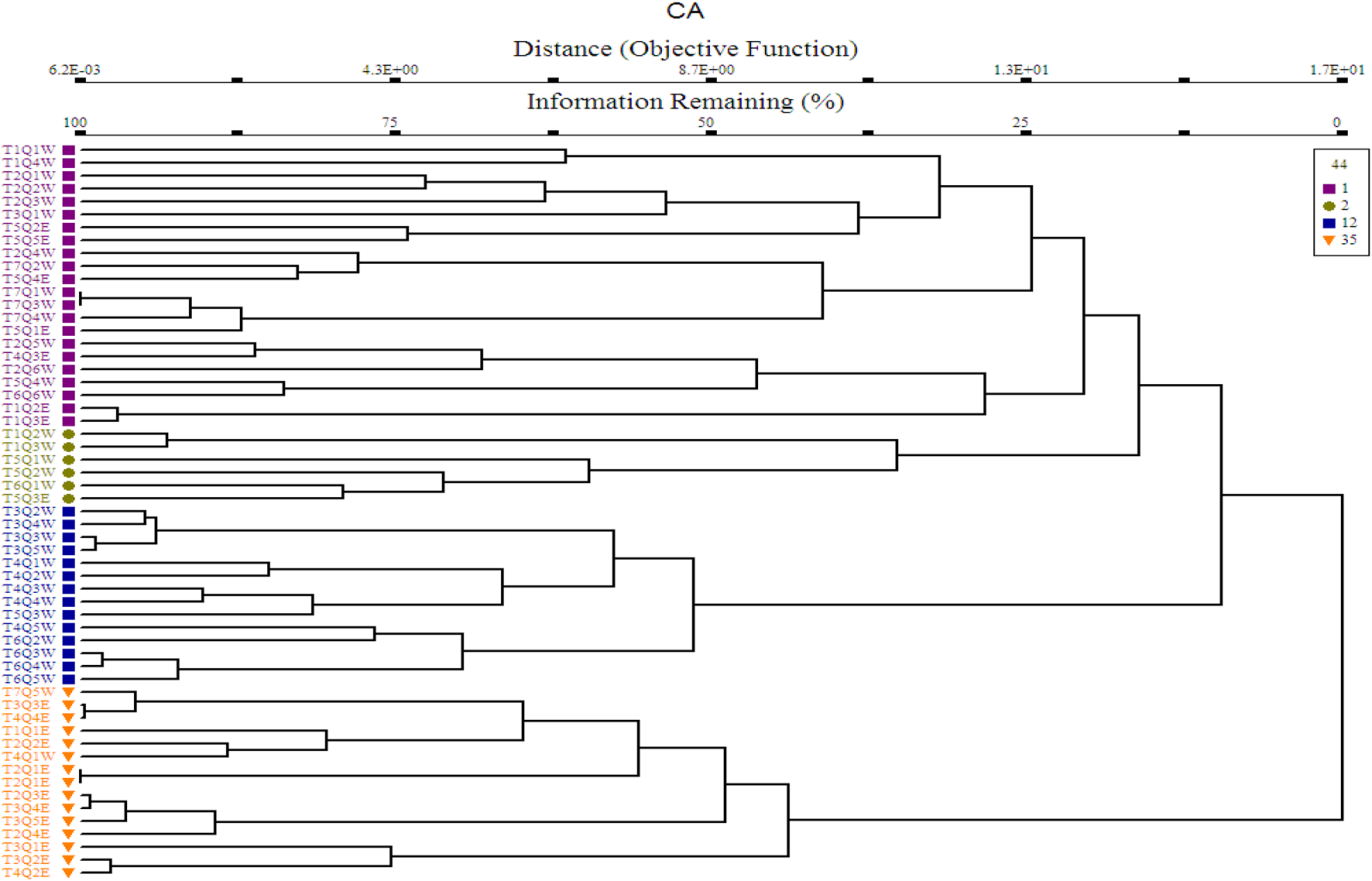
Cluster analysis classified all the stations quadrats into 4 major communities.

The result of cluster analysis classified all the quadrat into 4 major plant communities (Fig. 5). The detail description of each plant community are as follows:

#### *Pinus wallichiana-Berberis lycium-Impatiens brachycentra* plants community

**Figure 6:**
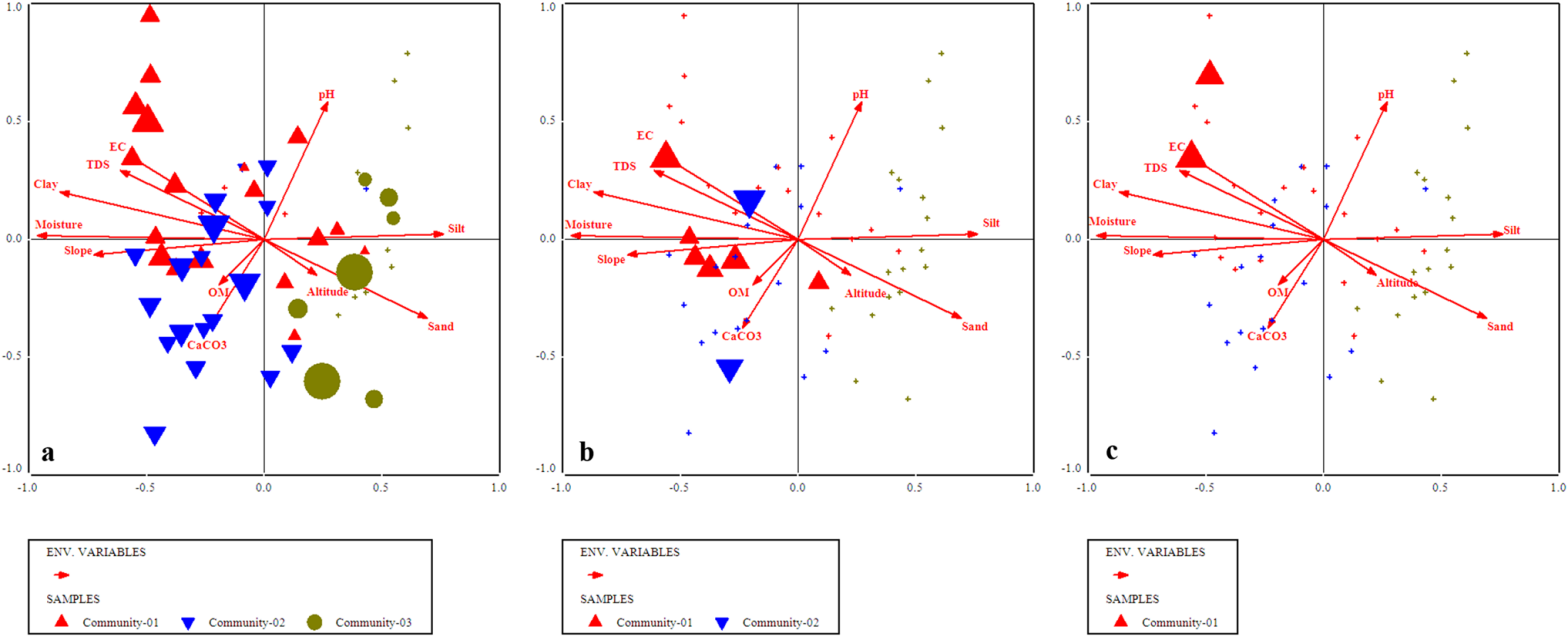
Indicator species biplot for (**a**) *Pinus wallichiana,* (**b**) *Berberis lyceum* and (**c**) *Impatiens brachycentra* top 3 indicators of first plant community in relation to various environmental variables.

The community name is based on the topmost indicator species. This community was observed at an elevation range of 1666–2882 m. The topmost indicator species of this community were *Pinus wallichiana, Berberis lyceum* and *Impatiens brachycentra,* one each from tree, shrubs and herbs, respectively (Fig. 6). The identified indicator species were the indicators of low altitude, soil pH, silt contents, and high percentage of calcium carbonates, clay, EC, soil moisture, organic matter, TDS and slope angle, at significant level (p ≤ 0.005).

*Abies pindrow, Rhus javanica, Quercus dilatata* were the dominant, while *Prunus persica, Diospyros lotus, Pistacia chinensis* were the rare tree species of this community*.* The dominant shrub species having high IVI of this community were *Viburnum grandiflorum, Berberis lyceum,* while the rare shrubs were *Rubus ulmifolius, Rubus occidentalis, Zanthoxylum armatum,.* The rare herb species of this community were *Dryopteris ramosa, Bistorta amplexicaulis, Arisaema flavum,* while *Eulaliopsis binata, Solidago virga-aurea* and *Galinsoga parviflora* were the rare herb species.

Edaphic and environmental gradients of this community ranges as, pH = 4.91–6.8, EC = 43–687 (µS), TDS = 44–428 (ppm), CaCO_3_ = 4.04–16.12%, OM = 0.85–9.85%, moisture contents = 2–32%, silt=14–52%, sand = 2–52%, clay = 10–78%, slope = 113–168° (Supplementary Table S1).

#### *Quercus incana-Buxus wallichiana-Agrostis gigantea* (QBA) community

**Figure 7:**
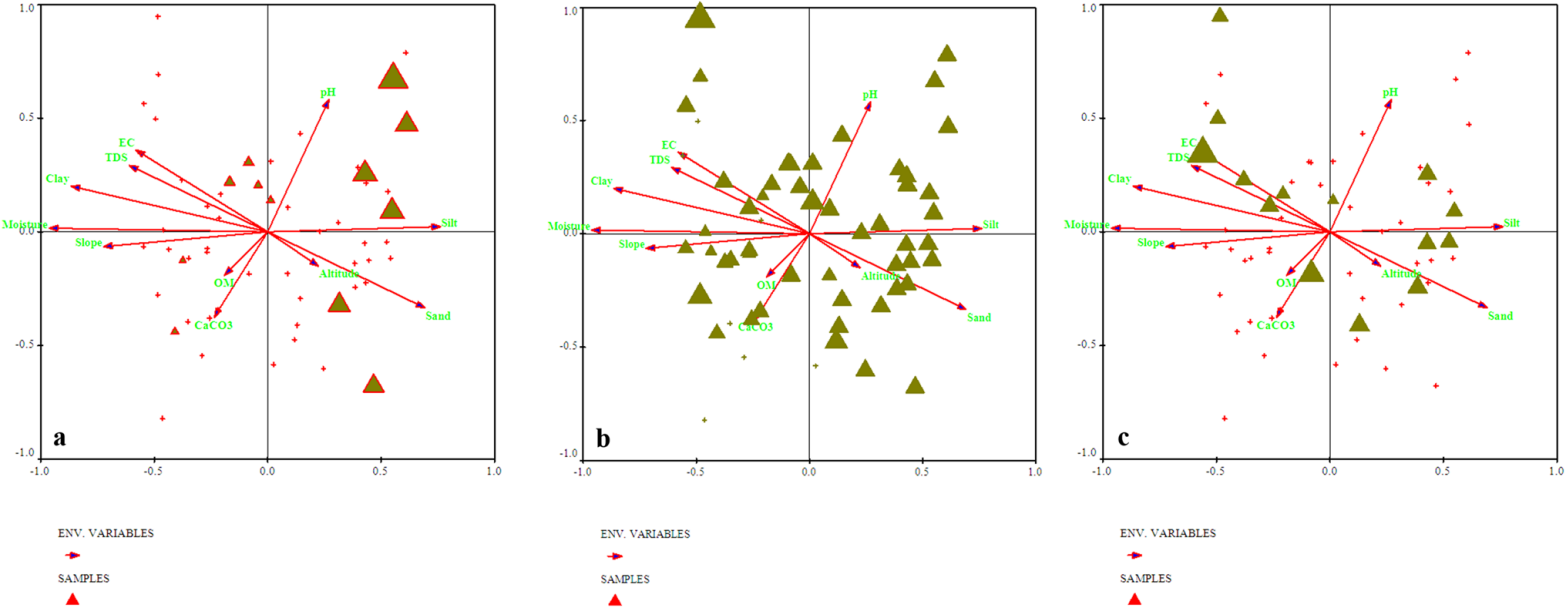
Indicator species biplot  for (**a**) *Quercus incana*, (**b**) *Boxus wallichiana* and (**c**) *Agrostis gigantea,* top 3 indicator plants of 2nd plants community after CCA of Canoco software.

This community was observed at an elevation range of 1824–2258 m. The topmost indicator species of this community were *Quercus incana, Boxus wallichiana* and *Agrostis gigantea* (Fig. 7)*.* The indicator species observed in this community were clustered around acidic pH, low silt, clay contents, calcium carbonates, moderate amount of organic matter, high level of TDS, EC, moisture contents and high slope angle at significant level (p ≤ 0.005). The values of edaphic and environmental gradients of this community ranges as, pH = 3.9–7.8, EC = 23–487 (µS), TDS = 41–288 (ppm), CaCO_3_ = 5.36–22.13%, OM = 3.10–12.1%, moisture contents = 7–39%, silt = 18–58%, sand = 3–56%, clay = 11–48%, slope = 110–1156^°^.

#### *Abies pindow*-*Rubus fruticosus*-*Poa annua* (ARP) community

**Figure 8:**
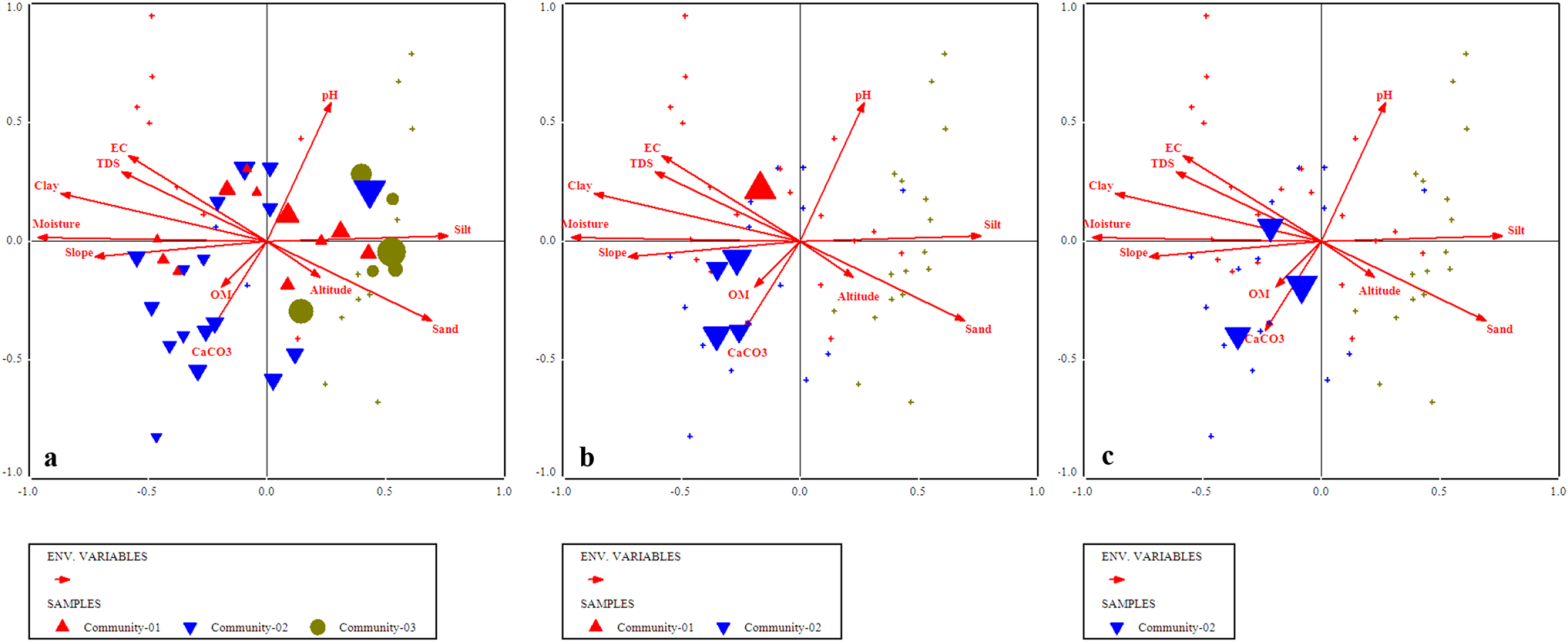
Indicator species biplots for (a) *Abies pindrow, *(b) *Rubus fruticocus* and (c) *Poa annua* in relation to various environmental factors.

The indicator species of this community were *Abies pindrow*, *Rubus fruticocus* and *Poa annua* (Fig. 8). The elevation range of this community was 2246–2824 m. The indicator species observed in this community have a significant positive relation with altitude, EC, less amount of CaCO_3_, moderate amount of clay, silt contents, moisture, neutral soil pH and less slope angle, while it was observed that these indicators were negatively correlated with the percentage of organic matter and TDS (p ≤ 0.005).

The dominant tree species having higher IVI were *Rhus javanica* and *Quercus dilatata*, while the tree species having lower IVI included *Cedrus deodara*, *Pistacia chinensis subsp. integerrima, Taxus baccata.* The abundant shrub species of this community having higher IVI were *Viburnum grandiflorum, Parrotiopsis jacquemontiana* while the shrub species were *Crataegus songarica, Berberis asiatica, Isodonrugosus*, *Viburnum cotinifolium*. The dominant herb species of this community were *Urtica dioca, Carex cardiolepis,* while the rare herb species of this community were *Achyranthes aspera, Tussilago farfara*, and *Adiantum venustum.* Edaphic and environmental gradients of this community ranges as, pH = 4.7–7.6, EC = 72–380 (µS), TDS = 51–239 (ppm), CacO_3_ = 6.36–19.13%, OM = 3.81–10.1%, moisture contents = 5–29%, silt = 16–54%, sand = 6–36%, clay = 13–67%, slope = 118–164° (Supplementary Table S1).

#### *Taxus baccata-Rubus ellipticus-Potentilla atrorubens* (TRP) community

**Figure 9:**
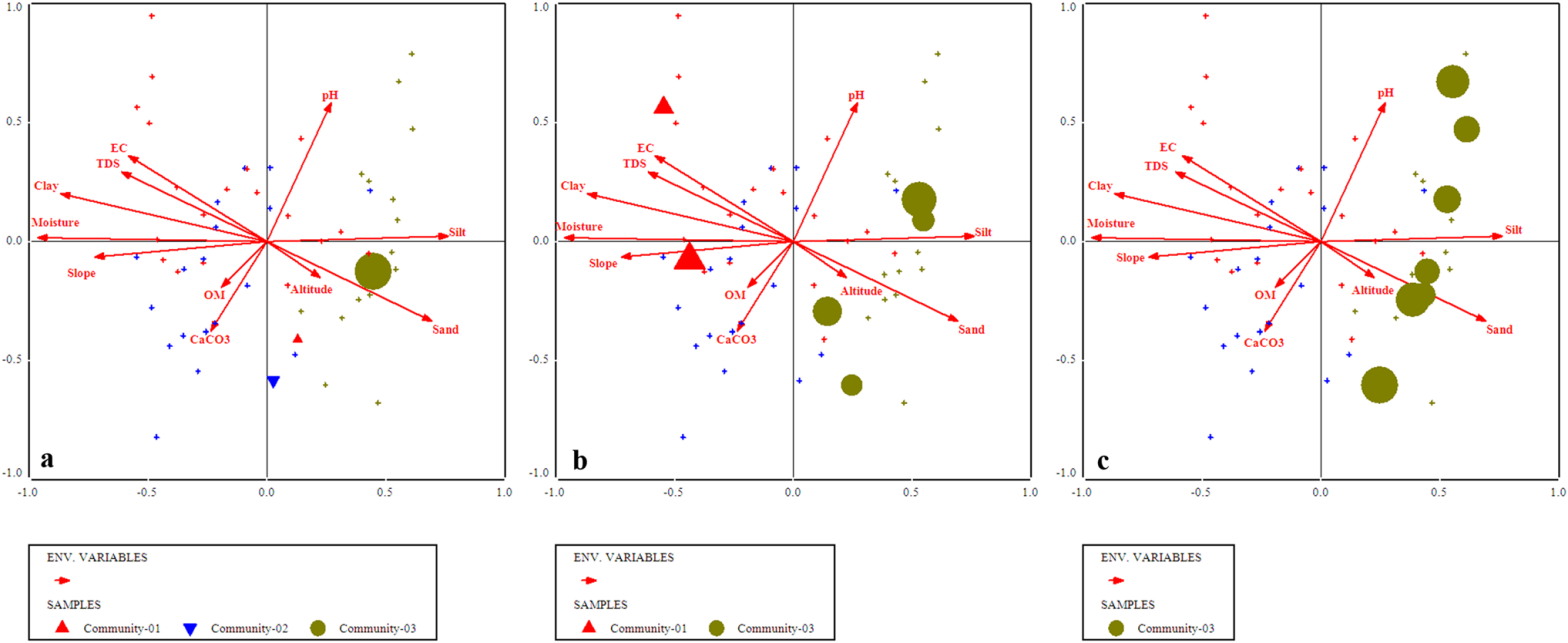
Indicator species biplots for (**a**) *Taxus baccata,* (**b**) *Rubus ellipticus* and (**c**) *Potentella atrorubens* top three indicators of 4th plants community.

The indicator species of this community were *Taxus baccata, Rubus ellipticus and Potentella atrorubens* ranging at the altitude of 2200–3028 m (Fig. 9). The indicator species observed in this community are clustered in relation to the lower altitude, less amount of calcium carbonate, clay contents, slope angle, TDS, higher pH, silt contents, moderate moisture and EC at p ≤ 0.005.

The dominant tree species of this community was *Abies pindrow*, while the rare tree species having lower IVI were *Cedrus deodara* and *Quercus dilatata.* The dominant shrub species of this community was *Viburnum grandiflorum,* while the rare shrub species was *Isodonrugosus.* The dominant herb species of this community were *Bistorta amplexicaulis, Muhlenbergia duthieana, Dryopteris ramosa,* while the rare herb species of this community were *Diplazium esculentum*, *Tussilago farfara*, and *Dryopteris marginali*.

The edaphic and environmental gradients of this community ranges as, pH = 5.1–7.2, EC = 53–403 (µS)s, TDS = 49–269 (ppm), CaCO_3_ = 2–18%, OM = 1.7–9.65%, moisture contents = 1–8%, silt = 23–68%, sand = 8–61%, clay = 10–41%, slope = 109–146° (Supplementary Table S1).

### Canonical Correspondence Analysis (CCA)/species ordinations under the influence of environmental gradients

**Figure 10:**
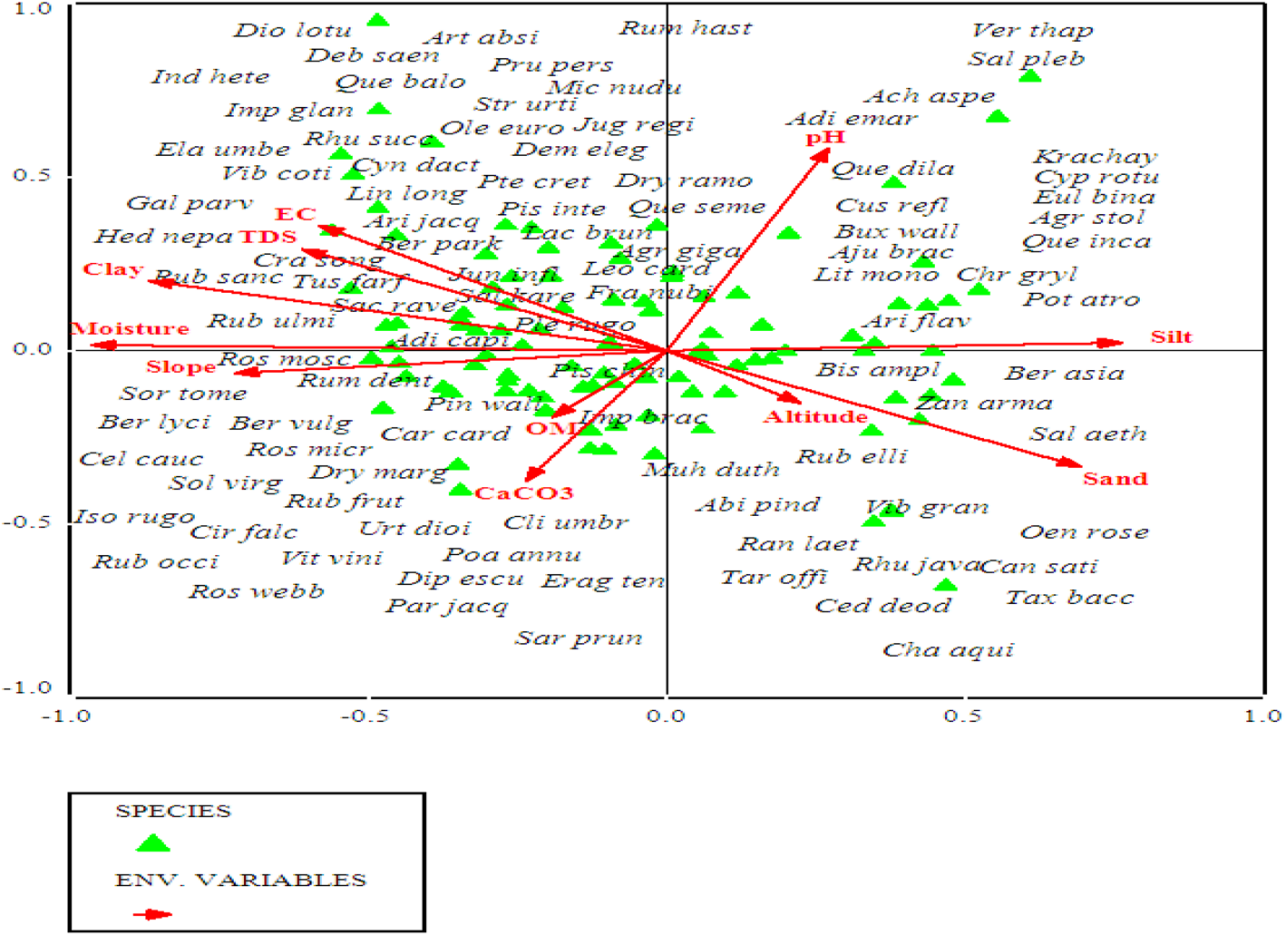
Canonical correspondence analysis (CCA) of 114 plant species in relation to measured environmental variables.

All the environmental variables including altitude, slope, aspect and edaphic variables i.e., organic matter, pH, CaCO_3,_ sand, silt and slay contents, soil moisture, EC and TDS significantly affecting the vegetation characteristics such as species abundance, distribution, and evenness in the studied area (p ≤ 0.001). Each border or cross in ordination bi-plot representing plant species in relation with environmental gradients, and the distance between them shows the differences index. The direction and length of arrows directed environmental variables in the CCA biplot, while the triangle shape indicated plant species based upon the relationship with environmental variables (Fig. 10). The length of the arrow indicated the dimension between different variables and degree of correlation between them. The variables occupying on the same axis shows direct relationship, while those on the opposite direction indicated an inverse relationship.

The first quadrant of CCA bi-plot shows that the plants including *Adiantum emarginatum*, *Achyranthes aspera*, *Quercus dilatata*, *Arisaema flavum*, *Buxus wallichiana* were primarily associated with soil pH and silt contents*.* Plant species i.e., *Debregea siasaeneb*, *Quercus baloot*, *Diospyros lotus*, *Rubus ulmifolius*, *Arisaema jacquemontii* etc. were mostly associated with EC, TDS, soil moisture and clay natured soil in the 2nd quadrant of canonical correspondence analysis. The plant species including *Berberis vulgaris, Dryopteris marginalis, Pinus wallichiana, Urtica dioca, Poa annua *etc were clustered around organic matter, calcium carbonate and slope angle at 3rd quadrant *.* The 4th quadrant of the CCA bi-plot shows the species that are associated with altitude of the mountain and sandy nature of the soil. These species were *Viburnum grandiflorum, Abies pindrow, Rhus javanica, Taraxacum officinale, and Zanthoxylum armatum *etc (Table 2; Fig. 10).

**Table 2:** Summary of canonical correspondence analysis of vegetation and environmental variables of the studied region.

Axes	1	2	3	4	TI
Eigenvalue	0.311	0.157	0.147	0.129	5.672
Species-environmental correlations:	0.917	3.823	2.832	0.836	
Cumulative percentage variance of species data:	5.5	8.3	10.8	13.1	
Of species environmental relation:	26..3	39.5	51.9	62.8	
Test of significance of first canonical: trace axis: eigenvalue	0.311				
F-value	2.671				
p-value	0.0002				
Test of significance of all canonical axis: trace	1.185	p-value	0.0010		
F-ratio	1.215

## Discussion

The vegetation structure, dynamics and distribution pattern are significantly influenced by environmental factors within and among ecosystems^71^. The present study reported 114 plant species, with maximum number of Rosaceae family, followed by Poaceae and Asteraceae. The above mentioned families are widely distributed across the globe having broad ecological amplitude that makes the possibility of their distribution particularly in microhabitats of temperate forest. These findings can be compared with the studies of Himalaya’s mountain where Rosaceae and Asteraceae were found as most dominant families^72^. The predominance of family Rosaceae may also be due to its central distribution from temperate to subtropical of the northern hemisphere. Our results are also comparable with the studies of Ilyas et al. & Amjad et al.^73,74^ reporting Poaceae and Ateraceae as the dominant families in Kabal Valley Swat, Valley and subtropical forest of Kotli Azad Kashmir, respectively. It was further observed that these families have characteristics similarities with the vegetation communities of Malakand, Hazara, Kashmir, and Gilgit Baltistan, Pakistan, where Asteraceae, Lamiaceae, Rosaceae, and Poaceae were reported as the uppermost prominent groups^53,75,76^.

The current study reported that the vegetation at higher altitudes contains typical moist temperate forest species, including *Pinus wallichiana*, *Abies pindrow*, *Viburnum grandiflorum* and *Bistorta amplexicaule,* which are in close harmony with the assemblage reported in the moist temperate Himalaya^77,78^. Among these *Pinus walllichiana*is the dominant tree in the region under consideration with highest IVI ( 1047), which corroborates with the findings of^74,79^ from western Himalayan region. The topography and altitude have negative relationship with the vegetation species richness and abundance, which tends to decrease along the altitude. Similarly Kharkwal et al.^80^, also reported the same pattern of species abundance and richness from Kuman, India. Our results are also in line with the findings of Bano et al.^58^, they also concluded that there was a decrease in numbers and diversity of species along the altitude in Beer hills, district Haripur, Khyber Pakhtunkhwa, Pakistan. The members of the family Poaceae were most common on the east slope compared to west slope due to more isolation, sandy nature of soil and less moisture contents, which provide a semi-xeric condition to the vegetation. Some xeric types of grasses like *Eulaliopsis binata *and *Muhlenbergia duthieana* were recorded as well on the same slope. The western slope of the mountain was dominated by some members of Gymnosperms such as *Pinus*, *Abies, Taxus* and moist temperate indicator species such as *Viburnum cotinifolium, Viburnum grandiflorum, Berberis lycium, Dryopteris ramosa, Urtica dioca *etc. These species are supported by the soil moist conditions, less availability of sunlight and more clay contents in soil. Various studies from semiarid region reported significant influence of microclimatic condition (such as air, soil temperature, evapotranspiration, wind speed along with edaphic factors (organic matter, soil moisture, soil depth and soil texture) on vegetation that subsequently results in distinct vegetation types inhabiting on different slope)^81^.

The attributes of plant species such as life form, leaf form (biological spectra), phenological trait mirrors the prevailing status of ecological and natural environmental condition of the region. The life form of a plant species is the reflection of adaptation to micro and macro climatic conditions and used frequently in vegetation assessment ^82^. In the current study, the dominant life form was hemicryptophytes (26%) followed by nanophanerophytes (26%) therophytes (20%) and mesophanerophytes (8%). These present findings show similarities  with Shimwell^83^ work as they reported hemicryptophytes as a dominant life form and considered as an indicator of the moist and cold temperate vegetation. A study was also carried out by Malik^84^ in Ganga Chotti and Badhori hills at Bagh district Azad Jammu Kashmir and reported that hemicryptophytes is the dominant life form in western Himalayas. Among the leaf form microphyll (33%) was the dominant most followed by leptophyll (24%) and mesophyll (16%). Our findings correlates with the studies of Khan et al.^85^ where they reported microphyll was dominant life form followed by leptophyll in moist temperate forest of Thandiani, Abbottabad, Khyber Pakhtunkhwa. Species with comparatively smaller leaves symbolize dry and cold climate, while larger leaves species occurs in warmer and moist climate. In current study microphyll were evident at higher altitude whereas leptophyll at lower elevation. Small leaves have been linked to cold or hot desert condition, which is an adaptive feature in preserving soil moisture. When the root is sensitive to low temperatures, which causes a decrease in water absorption from the soil, moisture retention is essential^86^. The large number of microphylls suggests a cool, sub-alpine, meadow climate with little moisture and nutrient absorption by the roots.

The total plant species in the study area were classified into 4 major plant communities through PC-ORD version 5. Bano et al.^58^ also identified 4 major plants communities in their study from Beer hills along the Indus River in district Haripur, Pakistan using the same statistical tool. Iqbal et al.^87^ conducted a study on Biha Valley, of Hindukush Mountain, District Swat, Pakistan and identified 7 different plant communities, using Two-way way cluster analysis classification. A phytosociological study was carried out by Ahmed^77^ in Himalayan forests of Pakistan and identified twenty-four different plant communities. The difference in number of the communities formed in any region might be due prevailing environmental conditions in those habitats.

The first plant community established at an elevation range of 1666–2882 m in the current study. The dominant species of this community were *Pinus wallichiana, Berberis lyceum,* and *Impatiens brachycentra*. It was influenced by environmental factors that ranges pH = 4.91–6.8, EC = 43–687 (µS), TDS = 44–428 (ppm), CaCO_3_ = 4.04–16.12%, OM = 0.85–9.85%, moisture contents = 2–32%, 14–52%, sand = 2–52, Clay = 10–78%, Slope = 113–168°. The dominant species of this community was influenced by EC, TDS, and clay content. Community 2 occurs at an elevation range of 1824-2258 m and dominated by *Quercus incana, Boxus wallichiana* and *Agrostis gigantea*. The dominant species have significant relationship with EC, TDS, Sand, pH, CaCO_3_, OM, clay content, sand and silt. Community 3 established at an elevation range of 2246–2824 m and dominated by *Abies pindrow*, *Rubus fruticocus* and *Poa annua*. The dominant species have strong relationship with soil pH, sand, altitude, CacO_3_ and OM. Community 4 established at the elevation range of 2200–3028 m and dominated by *Taxus baccata, Rubus ellipticus and Potentella atrorubens.* The dominant species of this community have strong relationship with pH, sand and altitude. Our finding of classification plant communities and identification of indicator species  corroborates with various research  studies in the adjacent mountainous^75,79,88–91^. Species richness and abundance is significantly influenced by the environmental variables including OM, TDS, OM, soil moisture and pH. Species abundance is positively correlated with environmental variable (OM, TDS, EC, Soil moisture) except soil pH. The direct correlation of quantitative characteristics of biodiversity with soil salinity, TDS and EC also reported by Canfora et al. & Liu et al.^92,93^. The moisture content significantly increases species richness and abundance, which are in line with the studied of Niu et al. ^94^ where species richness decreases by 3.56% with 10 degree decreases in the soil moisture. So, we can say that decreasing moistures also decreases in species richness.

We used CCA analysis to further confirm the significant relationship of different environmental variable (such as slope, topography, elevation, soil EC, TDS, moisture contents, soil texture, pH, CaCO_3_, OM etc.) with vegetation structure. The CCA bi-plot shows that most of the species influenced by EC, TDS, and CaCO_3_, slope, soil moisture contents and clay, silt, pH contents of the soil. These findings shows similarity with the study conducted, in Suez region of Egypt, that different physiographic and soil variables (such as latitude, longitude altitude pH, TDS, CaCO_3_, silt and sand) contributes to the distribution of vegetation pattern. The regression analysis among various environmental variables with species richness and abundance clearly revealed that the effect of a single environmental variables such as soil EC, TDS, CaCO_3,_ pH and moisture contents upon species abundance is steady, it means that the abundance of a species is likely affected by a specific environmental variable. While the richness of plant species affected by different variables in different ways due to various in demands and needs of the species. Similarly, Nadal-Romero et al.^95^, also conducted research and analyzed environmental factors (i.e., soil properties and aspect) and species diversity. Based upon these results from different regions of the world they concluded that not only species diversity is totally dependent on soil characteristics, but also on topography as well^81^. Slope and aspect are also known to affect the diversity and density of plant communities.

The studies reveal that *Pinus wallichiana *and *Viburnum grandiflorum,* are also the indicator species of moist temperate forest^87^. Furthermore, remaining species are somewhat native or exotic to the moist temperate forest ecosystem. While among the herbs and shrubs *Buxus wallichiana* (IVI = 568) and *Dryopteris ramosa* (IVI = 165) respectively were also founded abundant in the area. A similar study was also carried out by Haq et al.^58^, where they find out herbaceous vegetation as a dominant form in Beer Hills district Haripur, Khyber Pakhtunkhwa in the Himalayan range of Pakistan.

## Conclusion

It is concluded that the Yakhtangay Hills in the western Hindu-Himalayan series has a considerably rich phytodiversity. Soil organic matter, TDS, EC, CaCO_3_ and moisture contents showed a linear correlation (i.e. positive correlation) with species abundance, while inverse relationship was recorded between pH and plant species abundance. Similarly, a significant positive correlation exist between species richness and soil organic matter, calcium carbonate and moisture contents while significant inverse correlation exist between species richness and soil pH, TDS and EC in the studied mountainous region. *Pinus wallichiana*, *Viburnum grandiflorum*, *Berberis lycium*, and *Dryopteris ramosa* were recorded as an indicators of moist temperate ecosystem. CCA ordination analysis showed that all the measured edaphic and climatic variables have significant impact on the distribution pattern and composition of plant species in the studied region. Statistical tool and techniques used in the current study can be used for the classification of plant communities, identification of indicator plants, impact of environmental gradient on vegetation complexity and dynamics in any micro or macro habitat of the world. The dominant and rare species of the Yakhtangay Hills in the District Shangla can be declared a site for conservation priority as a micro-habitat representative of the Hindu Kush Himalayas ecotonal space.

## Supplementary Information

Supplementary Information.

## Data Availability

All data generated or analyzed during this study are included in this published article (and its Supplementary Information files).
