# Supplementary material for: Vegetation assessments under the influence of environmental variables from the Yakhtangay Hill of the Hindu-Himalayan range, North Western Pakistan
Source: Sci Rep. 2022 Dec 5;12:20973. doi: 10.1038/s41598-022-21097-4 (PMC9722792; doi:10.1038/s41598-022-21097-4)
Supplement: Supplementary file 1 — Supplementary Information. [file 41598_2022_21097_MOESM1_ESM.docx]

**Supplementary Tables**

**Table S1. Soil variables and environmental data**

| **Quadrat** | **PH** | **EC (milli Saimen/cm)** | **TDS** | **CaCO_3_ (%)** | **Organic matter (%)** | **Silt (%)** | **Sand (%)** | **Clay (%)** | **Altitude (feet)** | **Slope angle** |
| --- | --- | --- | --- | --- | --- | --- | --- | --- | --- | --- |
| T7Q1W | 6.26 | 273 | 181 | 8.64 | 9.1 | 20 | 6 | 74 | 6510 | 160˚Northward |
| T1Q2W | 5.94 | 385 | 243 | 10.56 | 5.65 | 26 | 3 | 71 | 6277 | 160˚Northward |
| T2Q3W | 5.72 | 687 | 428 | 10.223 | 2.2 | 28 | 4 | 68 | 6714 | 160˚Northward |
| T2Q2W | 5.42 | 318 | 208 | 10.68 | 0.85 | 20 | 2 | 78 | 6530 | 145˚Southward |
| T1Q3W | 6.07 | 299 | 253 | 9.77 | 9.15 | 34 | 14 | 52 | 6406 | 165˚Westward |
| T2Q1W | 5.21 | 287 | 229 | 8.64 | 7.7 | 14 | 4 | 82 | 6242 | 165˚Westward |
| T1Q1W | 4.91 | 262 | 120 | 11.82 | 6.7 | 16 | 6 | 78 | 6157 | 165˚Nortward |
| T1Q4W | 5.01 | 277 | 94 | 10.91 | 6.3 | 38 | 24 | 38 | 6856 | 134˚Eastward |
| T3Q5W | 5.09 | 177 | 119 | 12.05 | 4.8 | 54 | 23 | 23 | 7419 | 142˚Northward |
| T3Q2W | 4.9 | 74.1 | 50 | 10.46 | 3.75 | 36 | 36 | 28 | 6515 | 156˚Northward |
| T5Q4E | 6.75 | 437 | 285 | 10.05 | 9.15 | 29 | 6 | 65 | 6713 | 166˚Northward |
| T4Q4W | 5.5 | 362 | 231 | 11.5 | 8.35 | 37 | 8 | 55 | 6930 | 156˚Northward |
| T6Q2W | 6.1 | 339 | 228 | 16.56 | 8.9 | 26 | 12 | 62 | 6914 | 158˚Northward |
| T4Q3W | 5.3 | 341 | 215 | 19.13 | 7.9 | 38 | 6 | 56 | 6844 | 144˚Westward |
| T4Q2W | 5.23 | 385 | 234 | 14.11 | 10.1 | 28 | 9 | 63 | 6611 | 156˚Northtward |
| T3Q4W | 5.03 | 116 | 239 | 9.13 | 9.55 | 28 | 16 | 56 | 7216 | 161˚westward |
| T2Q6W | 4.8 | 371 | 231 | 9.56 | 9.05 | 19 | 22 | 59 | 7511 | 156˚westward |
| T4Q1E | 5.2 | 214 | 176 | 12.14 | 7.85 | 16 | 17 | 67 | 6115 | 144˚Northward |
| T2Q5W | 4.7 | 149 | 99 | 13.5 | 5.4 | 23 | 19 | 58 | 7206 | 134˚Northward |
| T3Q3W | 4.9 | 165.2 | 108 | 15.23 | 5.6 | 29 | 13 | 58 | 6813 | 164˚Northward |
| T5Q3E | 6.7 | 278 | 278 | 11.03 | 9.85 | 44 | 18 | 38 | 6611 | 168˚Westward |
| T2Q4W | 6.2 | 404 | 404 | 9.78 | 8.7 | 37 | 9 | 54 | 6960 | 143˚westward |
| T6Q3W | 6.3 | 271 | 271 | 12.57 | 7.1 | 35 | 8 | 57 | 7494 | 134˚westward |
| T6Q5W | 6.5 | 92 | 92 | 15.88 | 3.8 | 46 | 16 | 38 | 7888 | 158˚Northward |
| T6Q1W | 6.25 | 178 | 178 | 5.68 | 9.55 | 28 | 4 | 68 | 6530 | 158˚Northward |
| T6Q4W | 6.3 | 143 | 143 | 6.36 | 9.45 | 37 | 19 | 44 | 7641 | 162˚Northward |
| T5Q4W | 6.1 | 151 | 151 | 8.18 | 6.2 | 34 | 13 | 53 | 7887 | 156˚Northward |
| T5Q3W | 6 | 74 | 74 | 8.86 | 5.7 | 39 | 17 | 44 | 7411 | 133˚Northward |
| T4Q5W | 5.7 | 194 | 114 | 7.04 | 5.4 | 34 | 18 | 48 | 7456 | 128˚Southward |
| T5Q5E | 6.8 | 114 | 188 | 8.54 | 7.55 | 32 | 18 | 50 | 7124 | 152˚Northward |
| T7Q3W | 6.4 | 192 | 134 | 9.12 | 7.2 | 42 | 42 | 16 | 7240 | 144˚Eastward |
| T5Q1E | 6.52 | 188 | 94 | 13.24 | 5.75 | 30 | 32 | 38 | 6219 | 156˚Northward |
| T5Q2E | 6.6 | 107 | 44 | 16.12 | 5.15 | 52 | 23 | 25 | 6433 | 134˚Northward |
| T3Q2E | 6.3 | 145 | 112 | 15.88 | 4.2 | 49 | 41 | 10 | 6516 | 162˚Northward |
| T3Q3E | 6.4 | 163 | 124 | 9.77 | 8.35 | 45 | 42 | 13 | 6881 | 142˚Northward |
| T2Q2E | 6.1 | 60 | 39 | 10.14 | 7.85 | 23 | 61 | 16 | 6443 | 136˚Eastward |
| T1Q2E | 5.2 | 43 | 46 | 9.77 | 7.45 | 48 | 38 | 14 | 6613 | 128˚Westward |
| T3Q4E | 6.4 | 185 | 133 | 12.5 | 4.35 | 44 | 40 | 16 | 7216 | 118˚Northward |
| T6Q2W | 6.5 | 63 | 41 | 6.36 | 8.9 | 54 | 24 | 22 | 7190 | 134˚Eastward |
| T4Q4E | 7.2 | 103 | 67 | 7.56 | 5.95 | 50 | 28 | 22 | 6994 | 109˚Eastward |
| T3Q5E | 6.5 | 97 | 61 | 8.86 | 9.65 | 47 | 38 | 15 | 7519 | 118˚Eastward |
| T2Q3E | 6.2 | 92 | 69 | 10.91 | 3.1 | 44 | 23 | 33 | 6711 | 146˚Eastward |
| T4Q2E | 6.9 | 403 | 269 | 2.04 | 7.95 | 68 | 22 | 10 | 6416 | 141˚Northward |
| T4Q1W | 6.5 | 90 | 57 | 6.36 | 6.4 | 52 | 14 | 34 | 6311 | 134˚Southward |
| T1Q3E | 5.2 | 153 | 96 | 3.411 | 4.9 | 43 | 16 | 41 | 6856 | 135˚Southward |
| T1Q1E | 5.1 | 53 | 54 | 6.36 | 3.1 | 48 | 32 | 20 | 6019 | 117˚Eastward |
| T7Q5W | 4.9 | 79 | 50 | 8.86 | 1.7 | 47 | 32 | 21 | 7940 | 112˚southward |
| T2Q4E | 6.2 | 138 | 235 | 12.88 | 8.9 | 56 | 8 | 36 | 7244 | 141˚southward |
| T3Q1E | 6.2 | 69 | 49 | 12.05 | 7.1 | 45 | 44 | 11 | 6244 | 125˚southward |
| T4Q3E | 7.16 | 72 | 51 | 13.91 | 7.35 | 51 | 36 | 13 | 6732 | 118˚Northward |
| T2Q1E | 5.3 | 158 | 99 | 18.192 | 6.9 | 38 | 36 | 26 | 6113 | Northward |
| T7Q4W | 6.4 | 87 | 135 | 4.04 | 8.85 | 45 | 27 | 28 | 7530 | 142˚Southward |
| T5Q1W | 6.2 | 43 | 123 | 6.74 | 7.85 | 38 | 12 | 50 | 6411 | 123˚Eastward |
| T7Q2W | 6.3 | 91 | 144 | 11.15 | 5.4 | 42 | 48 | 10 | 6930 | 114˚southward |
| T5Q2W | 6.2 | 114 | 117 | 13.14 | 4.1 | 31 | 19 | 50 | 6823 | 113˚Northward |
| T3Q1W | 5.9 | 84 | 98 | 15.9 | 5.85 | 52 | 22 | 26 | 6316 | 142˚eatsward |

**Table S2. Floristic composition of the studied region**

| **Name of the plant** | **HABIT** | **Family** | **Class** | **Sub- Classes** |
| --- | --- | --- | --- | --- |
| *Strobilanthes urticifolia* Wall. ex Kuntze | Herb | Acanthaceae | Angiosperm | Dicot |
| *Achyranthes aspera* L. | Herb | Amaranthaceae | Angiosperm | Dicot |
| *Pistacia chinensis subsp. integerrima*(J.L.Stewart) Rech.f. | Tree | Anacardiaceae | Angiosperm | Dicot |
| *Pistacia integerrima* J.L.Stewart ex Brandis | Tree | Anacardiaceae | Angiosperm | Dicot |
| *Rhus javanica* auct. | Tree | Anacardiaceae | Angiosperm | Dicot |
| *Rhus succedanea var. himalaica* J.D. Hooker | Tree | Anacardiaceae | Angiosperm | Dicot |
| *Arisaema flavum* (Forssk.) Schott. | Herb | Araceae | Angiosperm | Monocot |
| *Arisaema jacquemontii* Blume. | Herb | Araceae | Angiosperm | Monocot |
| *Hedera nepalensis* K.Koch. | Shrub | Araliaceae | Angiosperm | Dicot |
| *Artemisia absinthium* L. | Shrub | Asteraceae | Angiosperm | Dicot |
| *Cirsium falconeri* (Hook.f.) Petr. | Herb | Asteraceae | Angiosperm | Dicot |
| *Galinsoga parviflora Cav.* | Herb | Asteraceae | Angiosperm | Dicot |
| *Lactuca brunoniana (DC.) Wall. ex C.B.Clarke.* | Herb | Asteraceae | Angiosperm | Dicot |
| *Solidago virga-aurea L.* | herb | Asteraceae | Angiosperm | Dicot |
| *Taraxacum officinale (L.) Weber ex F.H.Wigg.* | Herb | Asteraceae | Angiosperm | Dicot |
| *Tussilago farfara L.* | herb | Asteraceae | Angiosperm | Dicot |
| *Diplazium esculentum* (Retz.) Sw. | Herb | Athyriaceae | Pteridophytes |  |
| *Impatiens brachycentra* Kar. & Kir. | Herb | Balsaminaceae | Angiosperm | Dicot |
| *Impatiens glandulifera* Arn. | Herb | Balsaminaceae | Angiosperm | Dicot |
| *Berberis asiatica* Roxb. ex DC. | Shrub | Berberidaceae | Angiosperm | Dicot |
| *Berberis lycium* Royle. | Shrub | Berberidaceae | Angiosperm | Dicot |
| *Berberis parkeriana* C.K.Schneid. | Shrub | Berberidaceae | Angiosperm | Dicot |
| *Berberis vulgaris* L. | shrub | Berberidaceae | Angiosperm | Dicot |
| *Lindelofia longiflora* (Benth.) Baill. | Herb | Boraginaceae | Angiosperm | Dicot |
| *Buxus wallichiana* Baill. | Shrub | Buxaceae | Angiosperm | Dicot |
| *Sarcococca pruniformis* Lindl. | Herb | Buxaceae | Angiosperm | Dicot |
| *Caesalpinia decapetala var. pubescens* (Tang & F.T*.* Wang) X.Y. Zhu | Shrub | Caesalpiniaceae | Angiosperm | Dicot |
| *Cannabis sativa* L. | Herb | Cannabaceae | Angiosperm | Dicot |
| *Viburnum cotinifolium* D. Don. | shrub | Caprifoliaceae | Angiosperm | Dicot |
| *Viburnum grandiflorum* Wall. ex DC. | shrub | Caprifoliaceae | Angiosperm | Dicot |
| *Cuscuta reflexa* Roxb. | shrub | Cuscutaceae | Angiosperm | Dicot |
| *Carex cardiolepis* Nees. | Herb | Cyperaceae | Angiosperm | Monocot |
| *Cyperus rotundus* L. | Herb | Cyperaceae | Angiosperm | Monocot |
| *Dryopteris marginalis* (L.) A. Gray. | herb | Dryopteridaceae | Pteridophytes |  |
| *Dryopteris ramosa* (C. Hope) C. Chr. | HERB | Dryopteridaceae | Pteridophytes |  |
| *Diospyros lotus* L. | Tree | [Ebenacea](http://www.efloras.org/florataxon.aspx?flora_id=5&taxon_id=10293) | Angiosperm | Dicot |
| *Elaeagnus umbellata* Thunb. | Shrub | Elaeagnaceae | Angiosperm | Dicot |
| *Quercus baloot* Griff. | Tree | Fagaceae | Angiosperm | Dicot |
| *Quercus dilatata* Tenore. | Tree | Fagaceae | Angiosperm | Dicot |
| *Quercus incana Bartram* Roxb. | Tree | Fagaceae | Angiosperm | Dicot |
| *Quercus semecarpifolia* Sm. | Tree | Fagaceae | Angiosperm | Dicot |
| *Parrotiopsis jacquemontiana* (Decne.) Rehder | Shrub | Hamamelidaceae | Angiosperm | Dicot |
| *Juglans regia L.* | Tree | Juglandaceae | Angiosperm | Dicot |
| *Juncus inflexus L.* | Herb | Juncaceae | Angiosperm | Monocot |
| *Ajuga bracteosa* Wall. ex Benth. | Herb | Lamiaceae | Angiosperm | Dicot |
| *Clinopodium umbrosum* (M.Bieb.) Kuntze. | Herb | Lamiaceae | Angiosperm | Dicot |
| *Leonurus cardiaca* L. | Herb | Lamiaceae | Angiosperm | Dicot |
| *Plectranthus rugosus* Wall. ex Benth | Shrub | Lamiaceae | Angiosperm | Dicot |
| *Salvia aethiopis* L. | Herb | Lamiaceae | Angiosperm | Dicot |
| *Salvia plebeia* R.Br. | Herb | Lamiaceae | Angiosperm | Dicot |
| *Litsea monopetala* (Roxb.) Pers*.* | Tree | Lauraceae | Angiosperm |  |
| *Olea europaea* L. | Tree | Oleaceae | Angiosperm |  |
| *Oenothera rosea* L'Hér. ex Aiton. | Herb | Onagraceae | Angiosperm | Dicot |
| *Desmodium elegans* (Lour.) Benth. | Shrub | Papilionaceae | Angiosperm | Dicot |
| *Indigofera heterantha* Brandis. | shrub | Papilionaceae | Angiosperm | Dicot |
| *Abies pindrow* (Royle ex D.Don) Royle | Tree | Pinaceae | Gymnosperm |  |
| *Cedrus deodara* (Roxb.) G.Don | Tree | Pinaceae | Gymnosperm |  |
| *Pinus wallichiana* A. B. Jacks. | Tree | Pinaceae | Gymnosperm |  |
| *Agrostis gigantea* Roth. | Herb | Poaceae | Angiosperm | Monocot |
| *Agrostis stolonifera* Leers. | Herb | Poaceae | Angiosperm | Monocot |
| *Chrysopogon gryllus* (L.) Trin. | herb | Poaceae | Angiosperm | Monocot |
| *Cynodon dactylon* (L.) Pers. | Herb | Poaceae | Angiosperm | Monocot |
| *Eragrostis tenella* (L.) P.Beauv. ex Roem. & Schult. | Herb | Poaceae | Angiosperm | Monocot |
| *Eulaliopsis binata* (Retz.) C.E.Hubb. | Herb | Poaceae | Angiosperm | Monocot |
| *Microstegium nudum* (Trin.) A.Camus. | herb | Poaceae | Angiosperm | Monocot |
| *Muhlenbergia duthieana* Hack. | Herb | Poaceae | Angiosperm | Monocot |
| *Poa annua* Cham. & Schltdl. | Herb | Poaceae | Angiosperm | Monocot |
| *Saccharum ravennae* (L.) L | Herb | Poaceae | Angiosperm | Monocot |
| *amplexicaulis* (D.Don) Greene. | Herb | Polygonaceae | Angiosperm | Dicot |
| *Rumex dentatus* L. | herb | Polygonaceae | Angiosperm | Dicot |
| *Rumex hastatus* D. Don. | Shrub | Polygonaceae | Angiosperm | Dicot |
| *Adiantum capillus-veneris* L. | Herb | Pteridaceae | Pteridophytes |  |
| *Adiantum caudatum* L. | Herb | Pteridaceae | Pteridophytes |  |
| *Adiantum emarginatum* D.C. Eaton. | Herb | Pteridaceae | Pteridophytes |  |
| *Adiantum venustum* D. Don. | Herb | Pteridaceae | Pteridophytes |  |
| *Pteris cretica* L. | Herb | Pteridaceae | Pteridophytes |  |
| *Rubus sanctus* auct. plur. | herb | Ranunculaceae | Angiosperm | Dicot |
| *Ranunculus laetus var. kashmiricus* Qureshi & Chaudhri | Herb | Ranunculaceae | Angiosperm | Dicot |
| *Crataegus songarica* auct. | Shrub | Rosaceae | Angiosperm | Dicot |
| *Fragaria nubicola* Lindl. | herb | Rosaceae | Angiosperm | Dicot |
| *Potentilla atrorubens* Rydb. | Herb | Rosaceae | Angiosperm | Dicot |
| *Prunus persica* (L.) Batsch | Tree | Rosaceae | Angiosperm | Dicot |
| *Rosa microphylla* Roxb. ex Lindl. | Shrub | Rosaceae | Angiosperm | Dicot |
| *Rosa moschata* Herrm. | Shrub | Rosaceae | Angiosperm | Dicot |
| *Rosa webbiana* Wall. | Shrub | Rosaceae | Angiosperm | Dicot |
| *Rubus ellipticus* Kupcsok. | Shrub | Rosaceae | Angiosperm | Dicot |
| *Rubus fruticosus auct.* [L.] | Shrub | Rosaceae | Angiosperm | Dicot |
| *Rubus occidentalis* Georgi. | shrub | Rosaceae | Angiosperm | Dicot |
| *Rubus ulmifolius* J.Presl & C.Presl. | shrub | Rosaceae | Angiosperm | Dicot |
| *Sorbaria tomentosa* (Lindl.) Rehder | Shrub | Rosaceae | Angiosperm | Dicot |
| *Zanthoxylum armatum* DC. | Shrub | Rutaceae | Angiosperm | Dicot |
| *Salix karelinii Turcz.* | Tree | Salicaceae | Angiosperm | Dicot |
| *Bergenia ciliata* (Royle) A.Br. ex Engl. | Herb | Saxifragaceae | Angiosperm | Dicot |
| *Verbascum thapsus* L. | Herb | Scrophulariaceae | Angiosperm | Dicot |
| *Taxus baccata* L. | Tree | Taxaceae | Gymnosperm |  |
| *Celtis caucasica* Willd. | Tree | Ulmaceae | Angiosperm | Dicot |
| *Chaerophyllum aquilegifolium* (All.) Koso-Pol. | Herb | Umbelliferae | Angiosperm | Dicot |
| *Debregeasia saeneb (*Forssk.) Hepper & J.R.I.Wood | Shrub | Urticaceae | Angiosperm | Dicot |
| *Urtica dioica* L. | Herb | Urticaceae | Angiosperm | Dicot |
| *Vitis vinifera subsp. sylvestris* (C.C.Gmel.) Hegi. | shrub | Vitaceae | Angiosperm | Dicot |
| *Asplenium bulbiferum* | Herb | Pteridaceae |  |  |
| [*Cyrtomium fortunei*](https://identify.plantnet.org/the-plant-list/species/Cyrtomium%20fortunei%20J.%20Sm./data) | Herb | Dryopteridaceae. | Pterediophytes |  |
| *Asplenium scolopendrium* | Herb | Aspleniaceae | Pterediophytes |  |

**Table S3. Plant communities in relation to different environmental variables**

| Quadrats | pH | EC | TDS | CaCO_3_ | OM | Moisture | Silt | Sand | Clay | Altitude | Slope | |
| --- | --- | --- | --- | --- | --- | --- | --- | --- | --- | --- | --- | --- |
| Community-01 | | | | | | | | | | | |  |
| T1Q1W | 4.91 | 262 | 120 | 11.82 | 6.7 | 24 | 16 | 6 | 78 | 6157 | 165 | |
| T1Q4W | 5.01 | 277 | 94 | 10.91 | 6.3 | 18 | 38 | 24 | 38 | 6856 | 134 | |
| T2Q1W | 5.21 | 287 | 229 | 8.64 | 7.7 | 25 | 14 | 4 | 82 | 6242 | 165 | |
| T2Q2W | 5.42 | 318 | 208 | 10.68 | 0.85 | 28 | 20 | 2 | 78 | 6530 | 145 | |
| T2Q3W | 5.72 | 687 | 428 | 10.223 | 2.2 | 27 | 28 | 4 | 68 | 6714 | 160 | |
| T3Q1W | 5.9 | 84 | 98 | 15.9 | 5.85 | 13 | 52 | 22 | 26 | 6316 | 142 | |
| T5Q2E | 6.6 | 107 | 44 | 16.12 | 5.15 | 16 | 52 | 23 | 25 | 6433 | 134 | |
| T5Q5E | 6.8 | 114 | 188 | 8.54 | 7.55 | 14 | 32 | 18 | 50 | 7124 | 152 | |
| T2Q4W | 6.2 | 404 | 404 | 9.78 | 8.7 | 18 | 37 | 9 | 54 | 6960 | 143 | |
| T7Q2W | 6.3 | 91 | 144 | 11.15 | 5.4 | 2 | 42 | 48 | 10 | 6930 | 114 | |
| T5Q4E | 6.75 | 437 | 285 | 10.05 | 9.15 | 31 | 29 | 6 | 65 | 6713 | 166 | |
| T7Q1W | 6.26 | 273 | 181 | 8.64 | 9.1 | 32 | 20 | 6 | 74 | 6510 | 160 | |
| T7Q3W | 6.4 | 192 | 134 | 9.12 | 7.2 | 9 | 42 | 42 | 16 | 7240 | 144 | |
| T7Q4W | 6.4 | 87 | 135 | 4.04 | 8.85 | 9 | 45 | 27 | 28 | 7530 | 142 | |
| T5Q1E | 6.52 | 188 | 94 | 13.24 | 5.75 | 13 | 30 | 32 | 38 | 6219 | 156 | |
| T2Q5W | 4.7 | 149 | 99 | 13.5 | 5.4 | 29 | 23 | 19 | 58 | 7206 | 134 | |
| T4Q3E | 7.16 | 72 | 51 | 13.91 | 7.35 | 5 | 51 | 36 | 13 | 6732 | 118 | |
| T2Q6W | 4.8 | 371 | 231 | 9.56 | 9.05 | 25 | 19 | 22 | 59 | 7511 | 156 | |
| T5Q4W | 6.1 | 151 | 151 | 8.18 | 6.2 | 22 | 34 | 13 | 53 | 7887 | 156 | |
| T6Q6W | 6.4 | 157 | 145 | 9.8 | 6.46 | 27 | 38 | 14 | 56 | 6219 | 157 | |
| T1Q2E | 5.2 | 43 | 46 | 9.77 | 7.45 | 6 | 48 | 38 | 14 | 6613 | 128 | |
| T1Q3E | 5.2 | 153 | 96 | 3.411 | 4.9 | 4 | 43 | 16 | 41 | 6856 | 135 | |
| Community-02 | | | | | | | | | | | | |
| T1Q2W | 5.94 | 385 | 243 | 10.56 | 5.65 | 29 | 26 | 3 | 71 | 6277 | 160 | |
| T1Q3W | 6.07 | 299 | 253 | 9.77 | 9.15 | 28 | 34 | 14 | 52 | 6406 | 165 | |
| T5Q1W | 6.2 | 43 | 123 | 6.74 | 7.85 | 30 | 38 | 12 | 50 | 6411 | 123 | |
| T5Q2W | 6.2 | 114 | 117 | 13.14 | 4.1 | 10 | 31 | 19 | 50 | 6823 | 113 | |
| T6Q1W | 6.25 | 178 | 178 | 5.68 | 9.55 | 28 | 28 | 4 | 68 | 6530 | 158 | |
| T5Q3E | 6.7 | 278 | 278 | 11.03 | 9.85 | 22 | 44 | 18 | 38 | 6611 | 168 | |
| Community-03 | | | | | | | | | | | | |
| T3Q2W | 4.9 | 74.1 | 50 | 10.46 | 3.75 | 15 | 36 | 36 | 28 | 6515 | 156 | |
| T3Q3W | 4.9 | 165.2 | 108 | 15.23 | 5.6 | 22 | 29 | 13 | 58 | 6813 | 164 | |
| T3Q4W | 5.03 | 116 | 239 | 9.13 | 9.55 | 27 | 28 | 16 | 56 | 7216 | 161 | |
| T3Q5W | 5.09 | 177 | 119 | 12.05 | 4.8 | 17 | 54 | 23 | 23 | 7419 | 142 | |
| T4Q1E | 5.2 | 214 | 176 | 12.14 | 7.85 | 28 | 16 | 17 | 67 | 6115 | 144 | |
| T4Q2W | 5.23 | 385 | 234 | 14.11 | 10.1 | 21 | 28 | 9 | 63 | 6611 | 156 | |
| T4Q3W | 5.3 | 341 | 215 | 19.13 | 7.9 | 23 | 38 | 6 | 56 | 6844 | 144 | |
| T4Q4W | 5.5 | 362 | 231 | 11.5 | 8.35 | 28 | 37 | 8 | 55 | 6930 | 156 | |
| T4Q5W | 5.7 | 194 | 114 | 7.04 | 5.4 | 20 | 34 | 18 | 48 | 7456 | 128 | |
| T5Q3W | 6 | 74 | 74 | 8.86 | 5.7 | 19 | 39 | 17 | 44 | 7411 | 133 | |
| T6Q2W | 6.1 | 339 | 228 | 16.56 | 8.9 | 29 | 26 | 12 | 62 | 6914 | 158 | |
| T6Q3W | 6.3 | 271 | 271 | 12.57 | 7.1 | 16 | 35 | 8 | 57 | 7494 | 134 | |
| T6Q4W | 6.3 | 143 | 143 | 6.36 | 9.45 | 26 | 37 | 19 | 44 | 7641 | 162 | |
| T6Q5W | 6.5 | 92 | 92 | 15.88 | 3.8 | 21 | 46 | 16 | 38 | 7888 | 158 | |
| Community-04 | | | | | | | | | | | | |
| T7Q5W | 4.9 | 79 | 50 | 8.86 | 1.7 | 8 | 47 | 32 | 21 | 7940 | 112 | |
| T1Q1E | 5.1 | 53 | 54 | 6.36 | 3.1 | 5 | 48 | 32 | 20 | 6019 | 117 | |
| T2Q1E | 5.3 | 158 | 99 | 18.192 | 6.9 | 8 | 38 | 36 | 26 | 6113 | 119 | |
| T2Q2E | 6.1 | 60 | 39 | 10.14 | 7.85 | 2.5 | 23 | 61 | 16 | 6443 | 136 | |
| T2Q3E | 6.2 | 92 | 69 | 10.91 | 3.1 | 2 | 44 | 23 | 33 | 6711 | 146 | |
| T2Q4E | 6.2 | 138 | 235 | 12.88 | 8.9 | 2 | 56 | 8 | 36 | 7244 | 141 | |
| T3Q1E | 6.2 | 69 | 49 | 12.05 | 7.1 | 4 | 45 | 44 | 11 | 6244 | 125 | |
| T3Q2E | 6.3 | 145 | 112 | 15.88 | 4.2 | 8 | 49 | 41 | 10 | 6516 | 162 | |
| T3Q3E | 6.4 | 163 | 124 | 9.77 | 8.35 | 4 | 45 | 42 | 13 | 6881 | 142 | |
| T3Q4E | 6.4 | 185 | 133 | 12.5 | 4.35 | 3 | 44 | 40 | 16 | 7216 | 118 | |
| T3Q5E | 6.5 | 97 | 61 | 8.86 | 9.65 | 5 | 47 | 38 | 15 | 7519 | 118 | |
| T4Q1W | 6.5 | 90 | 57 | 6.36 | 6.4 | 1 | 52 | 14 | 34 | 6311 | 134 | |
| T4Q2E | 6.9 | 403 | 269 | 2.04 | 7.95 | 7 | 68 | 22 | 10 | 6416 | 141 | |
| T4Q4E | 7.2 | 103 | 67 | 7.56 | 5.95 | 2 | 50 | 28 | 22 | 6994 | 109 | |

**Table S4. Total IVI of dominant and rare species**

| **Name of the plant** | **Total IVI** |
| --- | --- |
| *Pinus wallichiana* | 2366.951 |
| *Abies pindrow* | 1256.558 |
| *Quercus incana* | 549.0672 |
| *Rhus javanica* | 322.2602 |
| *Quercus dilatata* | 276.1245 |
| *Prunus persica* | 25.83333 |
| *Pistacia chinensis subsp. Integerrima* | 24.70185 |
| *Diospyros lotus* | 17.39903 |
| *Olea europaea* | 10.57527 |
| *Quercus baloot* | 4.301521 |
| *Viburnum grandiflorum* | 2118.09 |
| *Buxus wallichiana* | 1697.6 |
| *Cuscuta reflexa* | 663.206 |
| *Parrotiopsis jacquemontiana* | 211.043 |
| *Rosa moschata* | 152.059 |
| *Desmodium elegans* | 18.78548 |
| *Debregeasia saeneb* | 17.77269 |
| *Artemisia absinthium* | 12.92837 |
| *Rubus occidentalis* | 11.32238 |
| *Rubus ulmifolius* | 7.370525 |
| *Bistorta amplexicaulis* | 450.1136 |
| *Urtica dioica* | 422.2545 |
| *Dryopteris ramosa* | 366.2025 |
| *Arisaema flavum* | 337.0275 |
| *Impatiens glandulifera* | 23.47592 |
| *Juncus inflexus* | 13.20707 |
| *Salvia plebeian* | 12.11404 |
| *Verbascum thapsus* | 10.02554 |
| *Chaerophyllum aquilegifolium* | 8.795987 |
| *Solidago virga-aurea* | 7.769886 |
